# Comments from the editors on the term “standard of care”

**DOI:** 10.1111/jvim.16911

**Published:** 2023-11-09

**Authors:** Stephen P. DiBartola, Ken W. Hinchcliff

**Affiliations:** ^1^ Co‐editors‐in‐Chief, Journal of Veterinary Internal Medicine Englewood CO USA

According to an editorial (J Am Vet Med Assoc 252:1343‐1344, 2018) by Dr Gary Block, the term “standard of care” (SOC) is defined in veterinary tort law as “the standard of care required of and practiced by the average reasonably prudent, competent veterinarian in the community.” He goes on to say, “Although the SOC essentially represents the minimum acceptable level of care, there is much confusion surrounding the term, with the SOC frequently mischaracterized as equivalent to ‘best practices’.”

Historically, case management by an individual veterinarian has been judged in comparison to standards of practice by veterinarians in the local community. However, with widespread access of local veterinarians to continuing education and specialists, it is likely that “standard of care” will come to be evaluated based more on national than local standards. Another factor that complicates the issue is the fact that legally animals are considered property and owners can decide how much or how little to spend on care. Consequently, veterinarians often are faced with making a decision about providing care they know is suboptimal rather than no care when an owner has limited financial resources.

Dr Block concludes by saying, “Although the term itself is used frequently in journal articles and conference presentations, the profession, to a large extent, lacks any consensus on what constitutes SOC in clinical veterinary practice.”

In their article, “Benign ureteral obstruction in cats: Outcome with medical management,” Merindol et al (J Vet Int Med 37:1047‐1058, 2023) indicate that placement of a subcutaneous ureteral bypass (SUB) device is the “standard of care” for ureterolithiasis in cats. Rather, it seems likely the authors are using the term “standard of care” in the sense of “gold standard,” as described above by Dr Block.

The term “gold standard” has its origins in international finance where the value of a nation's currency is linked to a specific amount of gold. In medicine, it has come to refer to a method or procedure that is perceived to be the best available to diagnose or treat a particular disease. However, it can be challenging to identify a single “best” way to treat a particular disease because outcome may well depend upon the training, skill, and experience of the individual providing treatment. A procedure performed by someone with little training or experience could be expected to result in a worse outcome than would occur if performed by someone with extensive training and experience in the procedure.

The article by Merindol et al and Dr Aronson's letter have provided an opportunity for us as editors to reflect on the terms “standard of care” and “gold standard” of treatment.

